# Evaluation of Anterior Segment's Structures in Tilted Disc Syndrome

**DOI:** 10.1155/2016/5185207

**Published:** 2016-08-25

**Authors:** Ercan Ozsoy, Abuzer Gunduz, Ersin Ersan Demirel, Tongabay Cumurcu

**Affiliations:** ^1^Haseki Research and Training Hospital, Department of Ophthalmology, Istanbul, Turkey; ^2^Inonu University School of Medicine, Department of Ophthalmology, Malatya, Turkey

## Abstract

*Purpose*. To evaluate anterior segment's structures by Pentacam in patients with tilted disc syndrome (TDS).* Methods*. Group 1 included forty-six eyes of forty-six patients who have the TDS. Group 2 including forty-six eyes of forty-six cases was the control group which was equal to the study group in age, gender, and refraction. A complete ophthalmic examination was performed in both groups. All cases were evaluated by Pentacam. The axial length (AL) of eyes was measured by ultrasound. Quantitative data obtained from these measurements were compared between two groups.* Results*. There was no statistically significant difference for age, gender, axial length, and spherical equivalent measurements between two groups (*p* = 0.625, *p* = 0.830, *p* = 0.234, and *p* = 0.850). There was a statistically significant difference for central corneal thickness (CCT), corneal volume (CV), anterior chamber angle (ACA), and pupil size measurements between two groups (*p* = 0.001, *p* = 0.0001, *p* = 0.003, and *p* = 0.001). Also, there was no statistically significant difference for anterior chamber depth (ACD), anterior chamber volume (ACV), and lens thickness (LT) measurements between two groups (*p* = 0.130, *p* = 0.910, and *p* = 0.057).* Conclusion*. We determined that CCT was thinner, CV was less, and ACA was narrower in patients with TDS. There are some changes in the anterior segment of the eyes with tilted disc.

## 1. Introduction

The tilted disc syndrome (TDS) was first clearly described in 1944 [1]. The TDS is identified a nonhereditary bilateral condition that occurs equally in men and women [2]. A colobomatous oval disc is present because of superotemporal disc elevation with posterior displacement of the inferonasal disc [3]. This shape is accompanied by situs inversus of the retinal vessels, congenital inferonasal conus, thinning of the inferonasal retinal pigment epithelium and choroid, myopic astigmatism, posterior staphyloma or coloboma, and visual field defects. These features assumed result from a generalized ectasia of the inferonasal fundus that comprises the corresponding sector of the optic disc [4, 5].

The aetiology of the TDS is still controversial. Theories associated with the aetiology of the syndrome refer to the malclosure of the embryonic optic fissure, causing a coloboma of the inferior fundus or inferonasal hypoplasia of the optic nerve head [6].

Pentacam is a noncontact optical system specifically designed to image the anterior segment of the eye. Pentacam is an easy-to-use anterior segment analyzer, and its high reliability and repeatability and independence of the operator have been clearly documented [7]. Pentacam rotating Scheimpflug camera provides quantitative information and qualitative imaging of the anterior and posterior surfaces of the cornea, anterior chamber depth, anterior chamber angle, iris, and lens [8].

In this study, we evaluated anterior segment's structures by Pentacam and the axial length of eyes that were measured by ultrasound.

## 2. Materials and Methods

Our study was cross-sectional and case controlled. In the study, individuals were divided into two groups. The first group (group 1) included forty-six eyes of forty-six patients who have the TDS. Diagnosis of the TDS was made according to those features: inferonasal tilting, situs inversus, thinning of the inferonasal retinal pigment epithelium, and choroid and myopic astigmatism. The second group (group 2) including forty-six eyes of forty-six cases was the control group which was equal to the study group in age, gender, and refraction (spherical equivalent). Cases that have history of previous corneal disease, contact lens use, glaucoma, and trauma and underwent ocular surgery were excluded from the study.

Informed consent was obtained from TDS patients and control subjects, and the study was reviewed by the Ethics Committee. A complete ophthalmic examination was performed in both groups; it included uncorrected and best spectacle-corrected visual acuity (BSCVA) by the Snellen chart, cycloplegic refraction, applanation tonometry, slit lamp, and fundus inspection.

All cases were evaluated by Pentacam (Oculus Optikgeräte GmbH, Wetzlar, Germany). The measurements were made under dim light. Pentacam is based on a 180° rotating Scheimpflug camera which can take 12–50 single images to reconstruct the anterior chamber. After completing test, Pentacam software constructs a three-dimensional image of the anterior segment and calculates the anterior chamber parameters [9]. Three measurements were taken from each case by Pentacam. The images that were taken with Pentacam ensured measurements of central corneal thickness (CCT), corneal volume (CV), anterior chamber depth (ACD), anterior chamber angle (ACA), anterior chamber volume (ACV), lens thickness (LT), and pupil size. The axial length (AL) of eyes were measured by ultrasound (A-Scan, Biovision V Plus). A-Scan examinations were performed with an 8 MHz linear probe. Pentacam measurements were not affected by corneal deformation from the A-Scan probe because they were performed first. Proparacaine HCl 0.5% (Alcaine, Alcon, USA) was used for topical corneal anesthesia before the AL measurement. The AL measurements mean the distance from the anterior cornea to the retina. Both Pentacam and A-Scan measurements were made three times for every eye and the average of these three measurements was calculated. Quantitative data obtained from these measurements were compared between two groups.

### 2.1. Statistical Analysis

The SPSS software version 20.0 (SPSS Inc., Chicago, IL, USA) was used for statistical analysis. The Shapiro-Wilk test was used to determine normal distribution. *t*-test and Mann-Whitney *U* tests were used to compare the variables between the groups.

## 3. Results

The mean age of group 1 was 37.00 ± 16.03 years; the mean age of group 2 was 35.56 ± 11.64 years. The mean AL measurements of group 1 and group 2 were 23.50 ± 0.38 and 23.39 ± 0.41 mm, respectively. The mean spherical equivalent of group 1 and group 2 was −3.62 ± 1.75 and −3.69 ± 1.51 (Table 1).

The mean CCT of group 1 and 2 was 514.28 ± 39.74 and 541.78 ± 38.31 *μ*m, respectively. The mean CV of group 1 and 2 was 57.67 ± 4.57 and 60.92 ± 3.39 mm^3^, respectively; the mean ACA of group 1 and 2 was 29.55 ± 12.70° and 35.77 ± 5.810, respectively; the mean pupil size of group 1 and 2 was 4.59 ± 1.55 and 3.39 ± 1.23 mm, respectively (Table 2).

There was no statistically significant difference for age, gender, axial length, and spherical equivalent measurements between two groups (*p* = 0.625, *p* = 0.830, *p* = 0.234, and *p* = 0.850).

There was a statistically significant difference for CCT, CV, ACA, and pupil size measurements between two groups (*p* = 0.001, *p* = 0.0001, *p* = 0.003, and *p* = 0.001).

Also there was no statistically significant difference for ACD, ACV,and LT measurements between two groups (*p* = 0.130, *p* = 0.910, and *p* = 0.057).

## 4. Discussion

Congenital TDS appeared in 1-2% of population [10]. The TDS appears to originate from the incomplete closure of the embryonic fissure [11]. The TDS is a congenital optic disc anomaly that affects anterior and posterior segments of the eye [12]. In patients with TDS, it has been shown that there is a significant correlation between the abnormal shape of the optic disc and the abnormal shape of the cornea. It has been suggested that the factors that cause shaping of the optic disc can cause shaping of the cornea [13]. Jonas and Königsreuther showed a relation between the size of the optic disc and the cornea. They demonstrated a relation between wide cornea and optic disc [14]. It is shown that the abnormal shape and size of the optic disc are closely associated with the shape and size of the cornea. It is proved that there is a relation between CCT and the diseases which affect the posterior segment of the eye-like optic disc drusen, glaucoma, age related macular degeneration, retinal detachment, and diabetes mellitus [12]. In a study, it was reported that the mean CCT of patients with optic disc drusen was 601 *μ*m and the mean CCT of patients without drusen was 560 *μ*m. This result shows a developmental relationship between optic disc and corneal thickness [15]. Pakravan et al. [16] showed that CCT had an inverse correlation with optic disc area in glaucoma patients. In their study, the mean CCT was 528.7 *μ*m and the mean disc area was 2 mm^2^. Ornek and Ozdemir did not find a significant difference for CCT between patients with TDS and control group. In their study, the mean CCT was 547.5 *μ*m in TDS patients and 541.09 *μ*m in control group. They thought that there is not any correlation between CCT and the presence of TDS [12]. But, in our study, the mean CCT was 514.28 ± 39.74 *μ*m in TDS group and 541.78 ± 38.31 *μ*m in control group. In our study, CCT was thinner in patients with TDS to control group; therefore, corneal volume was less.

E. Chihara and K. Chihara [17] reported that the AL was longer and the index of ovalness was larger in eyes with TDS, which was the major cause of myopia in these patients. But, in our study, we did not find a difference between two groups for AL.

In a study which was made by A-Scan ultrasonography, Dehghani et al. [18] reported that LT was greater in TDS patients than control group; in their study, the mean LT was 4.10 mm in TDS patients and 3.83 mm in control group, but, in our study, the mean LT was 3.83 ± 0.46 mm in TDS patients and 3.67 ± 0.31 mm in control group and there was not a difference between two groups. In the same study, Dehghani et al. did not find a difference between two groups for ACD like our study. They found that the mean ACD was 3.44 mm in TDS group and 3.32 mm in control group [18] and we found 3.02 ± 0.33 mm and 3.13 ± 0.32 mm, respectively.

In this study, the mean pupil size was 4.59 ± 1.55 mm in patients with TDS and 3.39 ± 1.23 mm in control group. This difference was statistically significant. The pupil size may influence the outcomes of refractive surgeries due to its role in postoperative visual symptoms like glare and halo. The larger pupil size can also cause greater higher order aberrations (HOAs). Thus, the pupil size is an important factor to consider especially for those patients who are candidates for refractive surgery [19]. In our study, because pupil size in TDS patients was larger, we think that the patients who will undergo refractive surgery must carefully be evaluated for TDS preoperatively.

The refractive multifocal IOLs (MIOLs) have two or more circular zones with different diopter strength, every zone makes a focal point for objects in the near, intermediate, or far distance. The performance of the refractive MIOLs is affected by the pupil size because of the different foci in different refractive zones. The refractive MIOLs are pupil size dependent; they have the problems of glare and halos in a large pupil [20]. Therefore, we think that cases who are candidates for refractive MIOLs must be investigated for TDS before surgery.

In this study, the mean ACA was 29.55 ± 12.70° in TDS patients and 35.77 ± 5.81° in control group, the mean ACA was narrower in TDS patients, therefore we think that cases with TDS also must be followed due to primary angle closure glaucoma suspect in lifetime.

In conclusion, we determined that there may be some changes in the anterior segment of the eyes with tilted disc. Therefore, we think that it is necessary to investigate the anterior segment's structures of eyes with tilted disc in detail. In particular, the cornea must be investigated broadly. Thus, we think that will help in avoiding undesirable surprises which arise from the operations like refractive surgery. Also, the cases who are candidates for refractive MIOLs must be evaluated for TDS. This study must be made with greater number of patients, in different geographical areas and in different racial groups for better outcomes.

## Figures and Tables

**Table 1:** Demographic data, mean axial length, and spherical equivalent of groups.

	Group 1 (*n* = 46) (Mean ± SD)	Group 2 (*n* = 46) (Mean ± SD)	*p* value
Age (year)	37.00 ± 16.03	35.56 ± 11.64	0.625

Gender			
Male (*n*)	23 (%50.00)	22 (%47.80)	0.830
Female (*n*)	23 (%50.00)	24 (%52.20)	

AL (mm)	23.50 ± 0.38	23.39 ± 0.41	0.234
SE (D)	−3.62 ± 1.75	−3.69 ± 1.51	0.850

SE: spherical equivalent.

**Table 2:** The data of anterior segment obtained from Pentacam compared between groups.

Pentacam data	Group 1 (*n* = 46) (Mean ± SD)	Group 2 (*n* = 46) (Mean ± SD)	*p* value
CCT (*μ*m)	514.28 ± 39.74	541.78 ± 38.31	0.001^*∗*^
CV (mm^3^)	57.67 ± 4.57	60.92 ± 3.39	0.0001^*∗*^
ACD (mm)	3.02 ± 0.33	3.13 ± 0.32	0.130
ACA (°)	29.55 ± 12.70	35.77 ± 5.81	0.003^*∗*^
ACV (mm^3^)	182.54 ± 46.74	188.40 ± 28.94	0.910
LT (mm)	3.83 ± 0.46	3.67 ± 0.31	0.057
Pupil size (mm)	4.59 ± 1.55	3.39 ± 1.23	0.001^*∗*^

^*∗*^Statistically significant. CCT: central corneal thickness; CV: corneal volume; ACA: anterior chamber angle; ACD: anterior chamber depth; ACV: anterior chamber volume; LT: lens thickness.

## References

[1] Williams A., Williams A., Austen D. (2005). The tilted disc syndrome. *Practical Neurology*.

[2] Apple D. J., Rabb M. F., Walsh P. M. (1982). Congenital anomalies of the optic disc. *Survey of Ophthalmology*.

[3] Gündüz A., Evereklioglu C., Er H., Hepşen I. F. (2002). Lenticular astigmatism in tilted disc syndrome. *Journal of Cataract & Refractive Surgery*.

[4] Ciftci S. (2011). Unilateral tilted disc and ipsilateral keratoconus in the same eye. *BMJ Case Reports*.

[5] Vongphanit J., Mitchell P., Wang J. J. (2002). Population prevalence of tilted optic disks and the relationship of this sign to refractive error. *American Journal of Ophthalmology*.

[6] Vuori M.-L., Mäntyjärvi M. (2007). Tilted disc syndrome and colour vision. *Acta Ophthalmologica Scandinavica*.

[7] Barkana Y., Gerber Y., Elbaz U. (2005). Central corneal thickness measurement with the Pentacam Scheimpflug system, optical low-coherence reflectometry pachymeter, and ultrasound pachymetry. *Journal of Cataract and Refractive Surgery*.

[8] Rabsilber T. M., Khoramnia R., Auffarth G. U. (2006). Anterior chamber measurements using Pentacam rotating Scheimpflug camera. *Journal of Cataract and Refractive Surgery*.

[9] Cumurcu T., Sener S., Ozsoy E., Doganay S. (2012). Changes in anterior chamber parameters with the pentacam rotating scheimpflug and axial length measurements by ultrasound in patients who use isotretinoin. *Current Eye Research*.

[10] Arici K., Demircan S., Topalkara A., Güler C. (1997). Four sisters with tilted disc. *Journal of Retina-Vitreous*.

[11] Apple D. J. (1984). New aspects of colobomas and optic nerve anomalies. *International Ophthalmology Clinics*.

[12] Ornek K., Ozdemir M. (2008). Central corneal thickness in tilted disc syndrome. *Optometry and Vision Science*.

[13] Jonas J. B., Kling F., Grundler A. E. (1997). Optic disc shape, corneal astigmatism, and amblyopia. *Ophthalmology*.

[14] Jonas J. B., Königsreuther K. A. (1994). Macrodiscs in eyes with flat and large corneas. *German journal of ophthalmology*.

[15] Dohadwala A. A., Damji K. F. (2000). Familial occurrence of artefactual ocular hypertension from thick corneas and of primary open angle glaucoma in a French Canadian kindred. *Ophthalmic Genetics*.

[16] Pakravan M., Parsa A., Sanagou M., Parsa C. F. (2007). Central corneal thickness and correlation to optic disc size: a potential link for susceptibility to glaucoma. *British Journal of Ophthalmology*.

[17] Chihara E., Chihara K. (1994). Covariation of optic disc measurements and ocular parameters in the healthy eye. *Graefe's Archive for Clinical and Experimental Ophthalmology*.

[18] Dehghani C., Nowroozzadeh M. H., Shankar S., Razeghinejad M. R. (2010). Ocular refractive and biometric characteristics in patients with tilted disc syndrome. *Optometry*.

[19] Hashemian S. J., Soleimani M., Foroutan A. (2012). Ocular higher-order aberrations and mesopic pupil size in individuals screened for refractive surgery. *International Journal of Ophthalmology*.

[20] Wang I.-J., Hu F.-R. (2009). The new generation of diffractive multifocal intraocular lenses. *Journal of the Formosan Medical Association*.

